# Establishment of an In Vitro Scab Model for Investigating Different Phases of Wound Healing

**DOI:** 10.3390/bioengineering9050191

**Published:** 2022-04-28

**Authors:** Chao Liu, Helen Rinderknecht, Tina Histing, Jonas Kolbenschlag, Andreas K. Nussler, Sabrina Ehnert

**Affiliations:** Department of Trauma and Reconstructive Surgery, Siegfried Weller Research Institute, BG Unfallklinik Tübingen, University of Tübingen, Schnarrenbergstr. 95, D-72076 Tübingen, Germany; liuchao413812@gmail.com (C.L.); helen.rinderknecht@student.uni-tuebingen.de (H.R.); thisting@bgu-tuebingen.de (T.H.); jkolbenschlag@bgu-tuebingen.de (J.K.); sabrina.ehnert@med.uni-tuebingen.de (S.E.)

**Keywords:** in vitro scab model, HaCaT cells, keratinocytes, TGF-β, blood cells

## Abstract

Chronic wounds are a serious problem in clinical work and a heavy burden for individuals and society. In order to develop novel therapies, adequate model systems for the investigation of wound healing are required. Although in past years different in vitro and in vitro wound healing models have been established, a true human-like model does still not exist. Animal models are limited in their use due to species-specific differences in the skin, a lengthy manufacturing process, experimental costs, and ethical concerns. Both 2D and 3D in vitro models are usually comprised of only one or two skin cell types and fail to capture the reaction between blood cells and skin cells. Thus, our aim was to develop an in vitro scab model to investigate early reactions in the wound healing process. The here established scab model is comprised of HaCaT cells and freshly collected blood from healthy volunteers. The generated scabs were stably cultured for more than 2 weeks. TGF-β signaling is well known to regulate the early phases of wound healing. All three TGF-β isoforms and target genes involved in extracellular matrix composition and degradation were expressed in the in vitro scabs. To validate the in vitro scab model, the effects of either additional stimulation or the inhibition of the TGF-β signaling pathway were investigated. Exogenous application of TGF-β1 stimulated matrix remodeling, which loosened the structure of the in vitro scabs with time, also induced expression of the inhibitory Smad7. Inhibition of the endogenous TGF-β signaling, on the contrary, resulted in a rapid condensation and degranulation of the in vitro scabs. In summary, the here established in vitro scab model can be used to analyze the first phases of wound healing where blood and skin cells interact, as it is viable and responsive for more than 2 weeks.

## 1. Introduction

Chronic wounds impose a significant and often underappreciated burden to the individual, the healthcare system, and society. In Germany, more than 4.5 million people are treated for chronic wounds each year, resulting in costs of up to five billion Euros to the public health care system [1]. It is estimated that 1 to 2% of the population in developed countries will experience a chronic wound during their lifetime [2]. So, suitable models to study wound healing are needed for the investigation of treatment strategies.

In recent years, different in vitro and in vitro models for wound healing have been developed; however, there is not yet an ideal comprehensive model available that is close to the human situation. Different skin types, long production processes, high costs, and ethical aspects limit the use of animal models. At the same time, most of the in vitro models, even the more complex 3D models, were made with only one or two skin cell types [3]. Wound healing is a complex process that starts with the disruption of blood vessels and is followed by clot formation. The remaining phases of wound healing in the scab are defined by the interaction between skin and blood cells within the scab. This interaction, however, cannot be displayed in in vitro models that lack a blood cell compartment.

Therefore, with this study we aimed to establish an in vitro scab model that can be used to investigate the different phases of wound healing. In the early phases of wound healing the scab is comprised mainly of epidermal and blood cells. Considering these characteristics of wound healing, in our research, HaCaT cells, which are a human keratinocyte cell line, and fresh blood from healthy volunteers will be used to develop the in vitro scab model to mimic the early phases of wound healing. The clotted fibrin will serve as a 3D scaffold for this model system. It is expected that the entrapped immune cells will provide a large variety of cytokines to control the differentiation of the model system.

Wound healing is a dynamic process involving various growth factors, cytokines, and chemokines that is controlled by an equally complex signaling network. Transforming growth factor beta (TGF-β), which is released by platelets, monocytes, macrophages, and keratinocytes after injury, is of particular importance. It is involved in a multitude of wound healing processes, including the regulation of inflammation, the stimulation of angiogenesis, the proliferation of fibroblasts and keratinocytes, and the regeneration of the extracellular matrix (ECM). This includes the production and deposition of extracellular matrix proteins, e.g., connective tissue growth factor (CTGF), collagen, or fibronectin, but also ECM remodeling by matrix metalloproteinases (MMPs) [4,5]. For each of the three TGF-β isoforms, specific roles in wound healing have been described. TGF-β1 is a potent regulator of inflammatory responses and is therefore usually upregulated in the early phases of wound healing, mediating the switch from a pro- to an anti-inflammatory environment [6]. TGF-β1 and TGF-β2 are both involved in the recruitment of fibroblasts and immune cells from the circulation and the wound edges into the wounded area, thus promoting the formation of granulation tissue and collagen synthesis [6]. However, excessive or chronically elevated TGF-β1 may promote scar formation and fibrosis in adults. In contrast, TGF-β3 may reduce scarring in adults and even promote scarless healing in the fetus [7]. These examples show that TGF-β is a key regulator in the different phases of wound healing, affecting different cell types.

Therefore, TGF-β isoforms represent an ideal candidate to validate the established in vitro scab model: besides expression and secretion of TGF-β isoforms, the effects of exogenous TGF-β stimulation (recombinant human TGF-β1, TGF-β2, and TGF-β3) and inhibition (Alk5 inhibitor) on extracellular matrix production will be investigated.

## 2. Materials and Methods

### 2.1. Ethics

Blood donations to produce in vitro scabs were received from healthy volunteers with their written informed consent, following the Declaration of Helsinki (1964) and its most recent amendments. The study was approved by the local ethics committee with the number 844/2020BO2.

### 2.2. Culture of HaCat Cells

HaCaT cells were grown in MEM minimum media supplemented with 5% fetal calf serum (FCS). The medium was replaced every 3 to 4 days, and cells were passaged 1:10 to 1:20 when confluence was reached. Cells were used until passage 11.

### 2.3. Production and Culture of the In Vitro Scab Model

The addition of CaCl_2_ to the collected EDTA venous blood restored its coagulation in vitro (Protocol adapted from Pfeiffenberger et al. [3]). In brief, HaCaT cells were resuspended in coagulation medium (Dulbecco’s Modified Eagle’s Medium (DMEM)—high glucose, 7.5 mM CaCl_2_, 5% FCS) at a cell density of 0.5 to 1 × 10^6^ cells/mL. Fresh EDTA venous blood samples were obtained from a variety of donors and processed within 4 h for coagulation. Clots were made in sterile non-adherent 96-well plates with F-shaped bottom. A volume of 60 µL of coagulation medium was combined with 60 µL of blood and incubated for 1 h at 37 °C with 5% CO_2_. After coagulation, scabs were transferred to fresh 96-well-plates. To maintain nutrient supply and ideal growing conditions, a medium change was conducted every 48 h.

### 2.4. The Measurement of Size and Weight of the In Vitro Scab Model

At each time point, at least 5 scabs were transferred into new 96-well-plates. Measurements were done immediately to prevent drying of the scabs. Scab weights were determined using an analytical balance. Diameter/area were determined by microscopy using the ImageJ software. Images were taken in 1.25× magnification.

### 2.5. Resazurin Conversion

The mitochondrial conversion of the redox dye resazurin to resorufin was used to evaluate cell viability. In vitro scabs were transferred to fresh 96-well plates and rinsed with 100 µL PBS once. After addition of 100 µL resazurin solution (0.0025% resazurin in culture medium) scabs were incubated at 37 °C, protected from light. After 2 h, 50 µL of the cell-free supernatant were transferred to fresh 96-well-plates and the fluorescence intensity was immediately determined (Ex = 544 nm; Em = 590–10 nm). Blank values were subtracted as background [8].

### 2.6. Viability Staining

Scabs were stained in 100 μL of medium supplemented with 0.02 mM Calcein-AM for 10 min at 37 °C. Fluorescence microscopy images were recorded in the GFP channel immediately after incubation and at various magnifications (20 to 200-fold) depending on the experimental setup. ImageJ was used to analyze the images [9].

### 2.7. Cytokine Array

A cytokine profile from the conditioned culture medium on days 1, 2, 3, 5, and 7 of culture was determined using the RayBio^®^ Human Cytokine Array C5 (BioCat, Heidelberg, Germany). The array was performed according to the manufacturer’s instructions. Chemiluminescent signals were detected with a CCD camera (INTAS, Göttingen, Germany). ImageJ was used to quantify signal intensities. On each array membrane 6 spots (positive controls) were used for normalization.

### 2.8. Gene Expression Analyses

Total RNA was isolated by phenol–chloroform extraction and quantified using a spectrophotometer (Omega plate reader, BMG Labtech GmbH, Ortenberg, Germany). cDNA was synthesized using the First Strand cDNA Synthesis Kit from maximal 2500 ng total RNA (Thermo Fisher Scientific, Sindelfingen, Germany). Primer sequences and optimized RT-PCR conditions are summarized in Table 1. The products of RT-PCR were subjected to a 2% (*w*/*v*) agarose gel electrophoresis with ethidium bromide, and the results were analyzed by using ImageJ software. *18s* and *RPL13a* were used as an internal control for normalization.

### 2.9. Luciferase Reporter Assay

HaCaT cells were infected with the Smad1/4 reporter adenovirus (Ad5-BRE-Luc/provided from Prof. P. ten Dijke). Upon binding of phosphorylated Smad1/4 or Smad3/4, respectively, luciferase is expressed in the cytoplasm of the cells. Luciferase activity in cell lysates was measured according to the manufacturer’s instructions, using the SteadyGlo Luciferase Assay System (Promega, Madison, WI, USA). Results were normalized to total protein content.

### 2.10. Statistical Analysis

GraphPad Prism 8.01 was used to perform the statistical analysis. If not stated otherwise, data are presented as mean ± SEM. The number of independent experiments (biological replicates = N) and technical replicates (*n*) are noted for each experiment in the figure legends. Because a Gaussian distribution could not be assumed due to the small sample size, conditions were compared using non-parametric Kruskal–Wallis tests and Dunn’s multiple comparison tests. A *p* < 0.05 was considered as statistically significant.

## 3. Results

### 3.1. In Vitro Scabs Can Be Investigated for More than 14 Days in Culture

**Figure 1:**
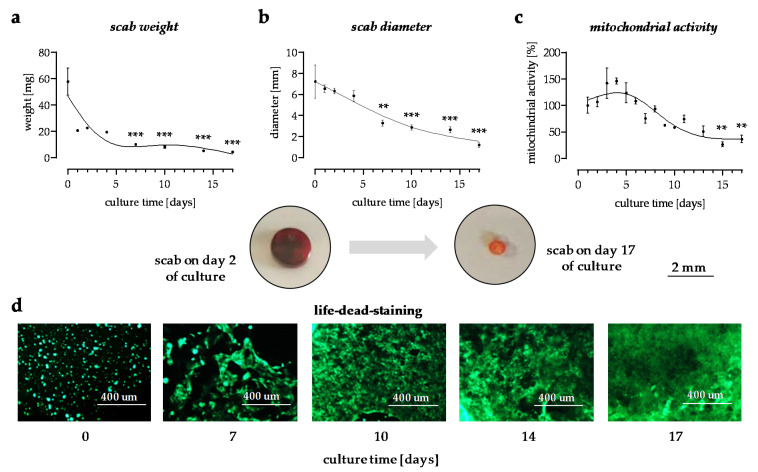
The in vitro scab model can be maintained in culture for more than 14 days. (**a**) The weight of the in vitro scabs in mg N = 4, *n* ≥ 5. (**b**) scab diameter in mm. N = 4, *n* ≥ 3. (**c**) Change in mitochondrial activity shown as fold of day 0. N = 3, *n* = 3. (**d**) Viability staining. Representative images were taken at a 10-fold magnification. Images were taken on days 0, 7, 10, 14, and 17 of culture. ** *p* < 0.01 and *** *p* < 0.001 as compared to day 0.

In this in vitro scab model, changes in the scab weight and scab volume were used to observe the stability on different time points of the culture. With culture time, the weight and volume of the scab continuously decreased (Figure 1a,b). The color of the scabs changed from dark brownish-red to pale red during the culture period. The viability of the cells within the scabs was monitored by changes in mitochondrial activity, measured by Resazurin conversion. The results show that mitochondrial activity increased during the first days, reaching a peak on the 4th day of culture, and then decreased over time. On day 14, mitochondrial activity was still detectable (Figure 1c). To confirm the results a viability staining with Calcein-AM was done. Representative images taken on days 7, 10, 14, and 17 of the culture showed that the number of viable HaCaT cells in the in vitro scabs increased with time (Figure 1d).

### 3.2. Cytokines Released from the In Vitro Scabs in the First Week of Culture

**Figure 2:**
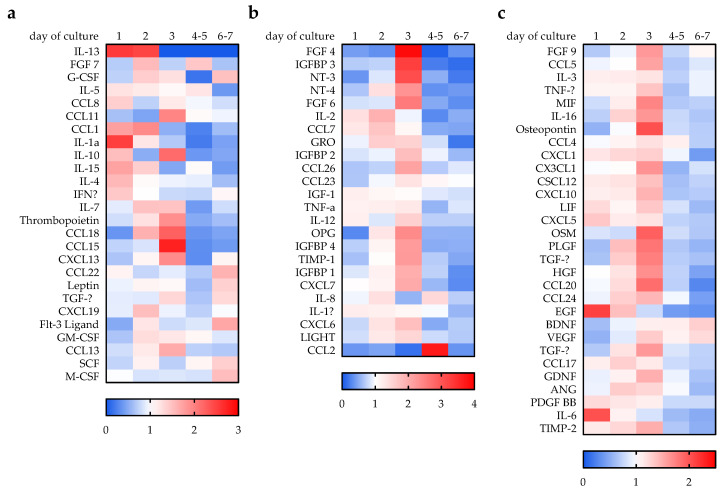
Cytokine profile from conditioned medium after 1, 2, 3, 5, and 7 days of culture. Conditioned medium from in vitro scabs (N = 4) was collected and a RayBio^®^ Human Cytokine Array C5 was performed (*n* = 2). Signal intensities were normalized to the positive controls on the membranes. (**a**–**c**) Data are presented as heat maps of the relative changes over time (signal intensities normalized to the average signal intensity of the individual cytokine). Cytokines were clustered in based on their expression profile over time.

The activity of the blood cells within the in vitro scabs was characterized by the cytokines released into the culture supernatant. A cytokine profile was determined from the conditioned medium after 1, 2, 3, 5, and 7 days of the culture using the RayBio^®^ Human Cytokine Array C5. The levels of a few cytokines, e.g., IL-1α, IL-4, IL-6, IL-13, IL-15, IFNγ, or EGF, were highest on day 1 of the culture and gradually decreased with time. A few cytokines, e.g., IL-2, CCL1, CCL7, or GRO, had the highest levels on day 2 of the culture. The levels of most cytokines increased until day 3 of the culture then gradually decreased. A few cytokines, e.g., SCF, M-CSF, BDNF, or CCL22, gradually increased with culture time (Figure 2).

### 3.3. All Three TGF-β Isoforms Can Be Detected in the In Vitro Scab Model

**Figure 3:**
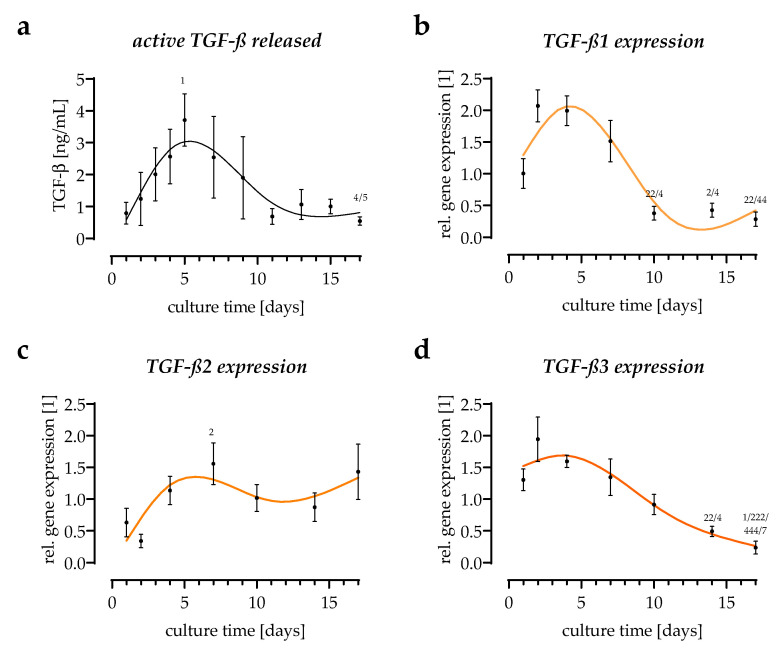
Expression and secretion of TGF-β over time. (**a**) Active TGF-β released from the in vitro scabs into the culture supernatants was detected with an adenoviral-based reporter assay. N = 4, *n* = 2. (**b**–**d**) Expression of the three TGF-β isoforms. Expression was determined at days 1, 2, 4, 7, 10, 14, and 17 of culture. N = 4, *n* = 2. ^X^ *p* < 0.05 and ^XX^ *p* < 0.01, and ^XXX^ *p* < 0.001 as compared to day X.

An adenoviral-based reporter assay, leading to the production of luciferase when Smad2/3-dependent TGF-β signaling is activated [10], was used to quantify the levels of active TGF-β released by the in vitro scabs into the culture supernatant. Released active TGF-β increased till day 5 of the culture and gradually declined after that (Figure 3a). To distinguish among the three TGF-β isoforms, semi-quantitative RT-PCRs were performed. *TGF-β1* expression changed the most, and its basal expression was highest among the three TGF-β isoforms. *TGF-β1* expression increased until day 4 of the culture, then declined. After 17 days of the culture, *TGF-β1* expression was lowest when compared to the other two isoforms. *TGF-β3* followed the same pattern as *TGF-β1*, but the changes were less prominent. In contrast, *TGF-β2* expression increased steadily throughout the entire culture period (Figure 3b).

### 3.4. Expression of Extracellular Matrix-Related Genes in the In Vitro Scabs

**Figure 4:**
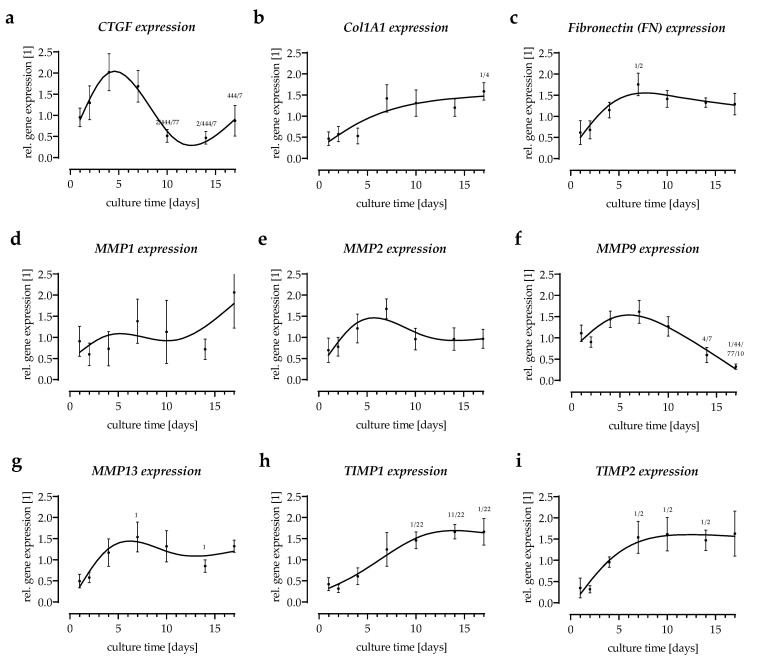
Expression of extracellular matrix-related genes in the in vitro scabs. Gene expression was determined by semi-quantitative RT-PCR on days 1, 2, 4, 7, 10, 14, and 17 of culture. Expression of target genes (**a**) *CTGF* (connective tissue growth factor), (**b**) *Col1A1* (collagen type 1 alpha-1), (**c**) *FN1* (fibronectin), (**d**–**g**) matrix metalloproteinases *MMP1*, *MMP2*, *MMP9*, and *MMP13*, (**h**,**i**) and tissue inhibitor of metalloproteinases *TIMP1* and *TIMP2* were determined. N = 4, *n* = 2. ^X^ *p* < 0.05, ^XX^ *p* < 0.01, and ^XXX^ *p* < 0.001 as compared to day X.

TGF-β1 is a multifunctional growth factor that exerts pleiotropic effects on wound healing. The target genes of TGF-β1, frequently described for wound healing, involve the production and degradation of the ECM [10]. In the in vitro scabs, expression of the early TGF-β-response gene *CTGF* showed the same trend as *TGF-β1* expression peaking around day 4 of the culture (Figure 4a). The expression of the extracellular matrix protein fibronectin (*FN1*) increased over the first week of culture in the in vitro scabs, then remained elevated (Figure 4b). The expression of collagen type I (*Col1A1*), which provides tensile strength to the skin in the wound, was progressively upregulated with culture time (Figure 4c). Matrix metalloproteases (MMPs) are proteases involved in matrix remodeling, which are present in both acute and chronic wounds. The expression levels of *MMP1*, *MMP2*, and *MMP13* showed a similar trend—their expression peaked around day 7 of the culture. The endogenous regulators of the MMP activity are the tissue inhibitors of metalloproteinases (TIMPs). The expression of both *TIMP1* and *TIMP2* increased in the in vitro scabs and reached a plateau after approx. 2 weeks of culture (Figure 4d–g).

### 3.5. TGF-β1 Could Promote the Area, Weight, and Mitochondrial Activity of the Scab Model

**Figure 5:**
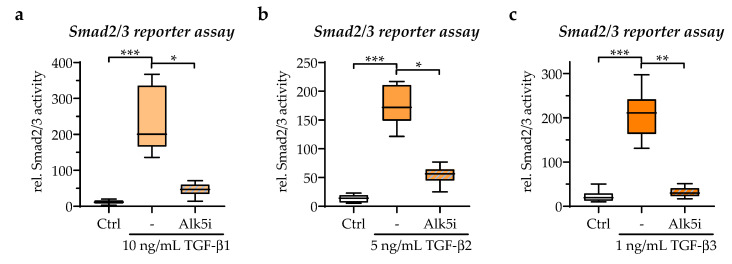
Exogenous stimulation with recombinant human TGF-β1-3 activates TGF-β signaling in the in vitro scabs. (**a**) 10 ng/mL of TGF-β1, (**b**) 5 ng/mL of TGF-β2, and (**c**) 1 ng/mL of TGF-β3 significantly induced TGF-β signaling in the in vitro scabs. TGF-β signaling was detected with an adenoviral-based reporter assay. The addition of 500 nM of ALK5i (SB431542) effectively blocked TGF-β signaling from all three TGF-β isoforms. Data are presented as box plots. Data were compared by non-parametric one-way ANOVA followed by Dunn’s multiple comparison test. * *p* = 0.05, ** *p* = 0.01, and *** *p* = 0.001.

The characterization of the established in vitro scab model revealed responses in TGF-β signaling and the expression of associated target genes. Considering the role of TGF-β in wound healing, the effect of exogenous applied TGF-β isoforms and the inhibition of TGF-β by Alk5i on the scab model were analyzed. With the Smad2/3 reporter assay, appropriate concentrations of TGF-β isoforms and ALK5i for the exogenous stimulation of in vitro scabs were determined/chosen. At the same time, the physiological TGFβ levels were also considered. So, the concentrations for recombinant human TGF-β1, TGF-β2, and TGF-β3, for exogenous stimulation were defined as 10 ng/mL, 5 ng/mL, and 1 ng/mL, respectively. A concentration of 500 nM of ALK5i was sufficient to block the activities of all three TGF-β isoforms (Figure 5).

**Figure 6:**
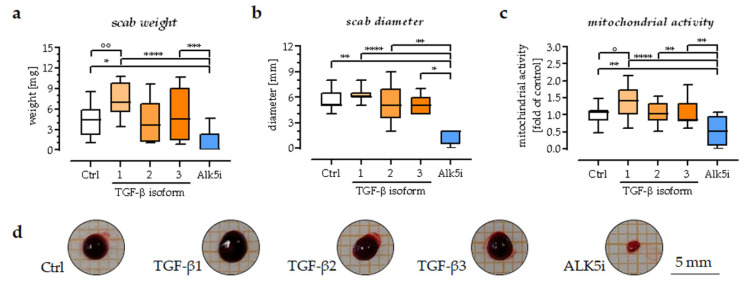
Effect of exogenous stimulation with recombinant human TGF-β isoforms and their inhibitor (Alk5i) on the weight, area, and mitochondrial activity of the in vitro scabs. (**a**) Weight of the in vitro scabs (**b**) Area of the in vitro scabs. (**c**) Differences in mitochondrial activity on day 14 of culture. N = 4, *n* = 3. (**d**) Representative image of the in vitro scabs on day 14 of culture. Data are presented as box plots. Data were compared by non-parametric one-way ANOVA followed by Dunn’s multiple comparison test. ° *p* = 0.05 and °° *p* = 0.01 as compared to Ctrl group. * *p* = 0.05, ** *p* = 0.01, *** *p* = 0.001, and **** *p* = 0.0001 as compared to Alk5i group.

Therefore, the in vitro scabs were incubated for 14 days with either the exogenous addition of one of the three TGF-β isoforms or Alk5i. The exogenous stimuli were refreshed every second day during culture. The scabs were analyzed on days 2, 7, and 14 of the culture. The stability and viability of the scab models were determined by measuring the scab volume, weight, and mitochondrial activity. The results show that inhibition of TGF-β signaling (Alk5i) accelerated the decrease in scab volume and weight observed with culture time. The small scabs remaining at day 14 of the culture in the inhibitor (Alk5i) group were palest in color. Furthermore, the scabs in the inhibitor (Alk5i) group had the lowest mitochondrial activity. Exogenous stimulation with TGF-β1 caused opposite results to the inhibitor (Alk5i) group. The scabs in this group had the highest weight, volume, and mitochondrial activity. Exogenous stimulation with TGF-β2 and TGF-β3 had no significant effect on scab volume, weight, or mitochondrial activity (Figure 6).

### 3.6. Exogenous Stimulation with TGF-β1 Increased the Expression of Smad7 in the In Vitro Scabs

**Figure 7:**
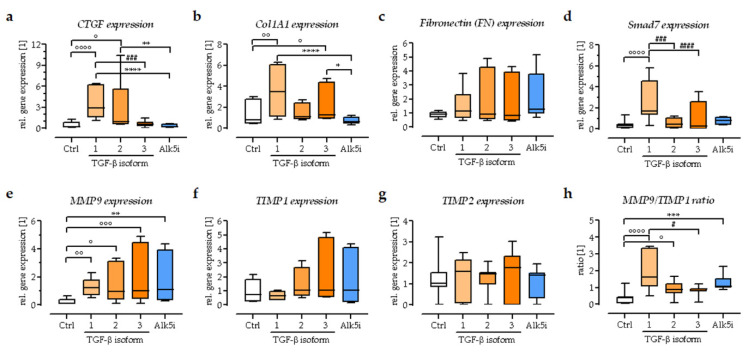
The expression of different ECM-related genes in the in vitro scabs. Gene expression was analyzed on day 14 of culture, normalized to the housekeeping genes, and represented as relative expression levels: (**a**) *CTGF* (connective tissue growth factor), (**b**) *Col1A1* (collagen type 1 alpha-1), (**c**) *FN1* (fibronectin), (**d**) inhibitory *Smad7*, (**e**) matrix metalloproteinases *MMP9*, (**f**,**g**) tissue inhibitor of metalloproteinases *TIMP1* and *TIMP2* were determined, (**h**) and the ratio of expressed *MMP9* and *TIMP1*. N = 4, *n* = 3. Data are presented as box plots. Data were compared by non-parametric one-way ANOVA followed by Dunn’s multiple comparison test. ° *p* = 0.05, °° *p* = 0.01, °°° *p* = 0.001, and °°°° *p* = 0.0001 as compared to Ctrl group. * *p* = 0.05, ** *p* = 0.01, *** *p* = 0.001, and **** *p* = 0.0001 as compared to Alk5i group. ^#^ *p* = 0.05, ^###^ *p* = 0.001, and ^####^ *p* = 0.0001 as indicated.

The expression of TGF-β target genes in the in vitro scabs was analyzed by semi-quantitative RT-PCR after 14 days of exogenous stimulation with TGF-β1-3 or ALK5i (Figure 7). Expression of the direct TGF-β target genes *CTGF*, *Col1A1*, and *FN1* was increased following exogenous stimulation with TGF-β1. The effect of the other two TGF-β isoforms was not as pronounced. As expected, exogenous stimulation with TGF-β1 also induced expression of the inhibitors *Smad7*, as an auto-regulatory mechanism. Exogenous stimulation with all three TGF-β isoforms, but also the Alk5i, promoted the expression of *MMP9*. The expression of *TIMP1* and *TIMP2* was not significantly affected by the stimulation. However, considering the interplay between MMPs and TIMPs (MMP9/TIMP1 ratio), this suggests that matrix degradation is strongest in the TGF-β1 treated scabs.

## 4. Discussion

This study aimed at developing a simple but comprehensive model for investigating the early phases of wound healing, showing the interplay between skin and blood cells. For mimicking a wound, a co-culture of keratinocytes and blood cells was carried out.

Normal wound healing includes four sequential but overlapping phases: hemostasis (0–several hours after injury), inflammation (1–3 days), proliferation (4–21 days), and remodeling (21 days–1 year) [11]. The communication and interplay between many distinct cell types are required for effective repair, and this process is tightly organized and regulated at different cellular levels [12]. In this study, an in vitro scab model was established by mixing HaCaT cells, fresh EDTA blood, and CaCl_2_. The addition of CaCl_2_ saturated the EDTA, such that coagulation could occur, which simulates the hematoma or scab formation. The formed fibrin clot served then as a 3D carrier for the entrapped cells that included keratinocytes and blood cells in the scab model. With culture time, the weight and area of the scabs became smaller, while an increased amount of HaCaT cells was detected, suggesting that the in vitro scabs start to resorb and remodel like it is found in vitro [11]. It is expected that the amount of blood cells decreases in the in vitro scabs with culture time. The cytokine profile underlines this assumption as the levels of most cytokines released into the culture supernatant peaked within the first 3 days of culture then gradually decreased with culture time. Of note, almost all cytokines detected were still well detectable in the culture supernatant after 7 days of culture. This is in line with the fact that the in vitro scabs could be cultured for more than 2 weeks.

In normal wound healing, TGF-β is quickly released from blood cells, especially thrombocytes, and keratinocytes. The released TGF-β then promotes keratinocytes’ proliferation and migration, differentiation, and ECM production [6]. Active TGF-β was also released from the here presented in vitro scabs, especially in the first days of the culture. The release of active TGF-β and the expression of the three TGF-β isoforms were similar. This result is consistent with the literature, which showed a rapid increase in circulating active TGF-β following a wound [13]. As TGF-β is a key regulator in wound healing, the here presented in vitro scab model shall be able to sense alterations in this signaling pathway. Besides the regulation of inflammation, a key role of TGF-β during wound healing is the production of the ECM. During wound healing, the ECM is not just architectural support for the tissue, but it also critically regulates cellular behavior by controlling cellular adhesion, migration, proliferation, and differentiation [14]. Representative target genes of TGF-β1 involved in the production and degradation of the ECM, are *CTGF*, *Col1a1*, *FN*, *MMPs*, and *TIMPs*.

Among them, CTGF is an ECM-associated heparin-binding protein that binds directly to integrins [15]. In vitro experiments have shown that *CTGF* expression is induced by non-canonical TGF-β signaling, including Ras/MEK/ERK-MAPK [16]. In line with the literature, *CTGF* expression is comparable to the expression of TGF-β1, which rapidly increased within the first days of the culture in the in vitro scabs. Both TGF-β and its target CTGF may then induce the expression of ECM proteins, e.g., collagen or fibronectin, thus participating in the formation of granulation tissue in wounds [17]. The observation that TGF-β induces the expression of collagen and fibronectin is not exclusive for fibroblasts, but also holds for a large variety of other cell types [18,19]. Thus, it is not surprising that *Col1A1* and *FN* expression in the in vitro scabs increased with a timely delay to *TGF-β1* and *CTGF*. The altered expression of collagen in the different phases is associated either with chronic and non-healing wounds or with keloid and hypertrophic scar formation [20]. Thus, the in vitro scab model was tested by exogenously applying recombinant human TGF-β. The addition of TGF-β, especially TGF-β1, increased the expression of *CTGF* and *Col1A1* in the in vitro scabs. With the increased expression of these two ECM proteins, a faster resolution and condensation of the in vitro scabs could be expected. However, TGF-β1-stimulated in vitro scabs were largest in size after 14 days of culture and in vitro scabs cultured with the ALK5i were palest and smallest in size. One explanation may be the expression of *FN*, which supports re-epithelialization [21,22], and was tendentially highest expressed in the Alk5i group. Another explanation might be the strong induction of inhibitory Smad7 in the in vitro scabs upon TGF-β1 stimulation, indicating an auto-regulatory mechanism [23]. But it is also feasible that the degradation of the ECM, by MMPs and TIMPs [24], is affected.

MMP1, MMP9, and MMP13 are the major chemokine regulators during wound healing [25]. Of these three, basal expressions of *MMP9* were most prominent in the in vitro scabs. All MMPs investigated showed an increase in expression within the first days of the culture, especially when TGF-β signaling was activated by exogenous TGF-β1-3. In a zebrafish model, a direct interaction between MMP9 and canonical TGF-β signaling was described [26]. In this model addition of recombinant MMP9 activated TGF-β signaling, supposedly by the increased release and activation of the cytokine. This could explain why the inhibition of TGF-β signaling with the Alk5i also increased *MMP9* expression in the in vitro scabs within 14 days of culture. In the human body, TIMPs bind to the active center of MMPs to form MMP-TIMP complexes in a 1: 1 ratio. This process blocks the binding of MMPs to ECM substrates, effectively inhibiting the activity of MMPs [27]. Among the TIMPs, both TIMP1 and TIMP2 may inhibit MMP9 [28]. TIMP1 is expressed in the epithelial cells (e.g., HaCaT cells) and fibroblasts of healing excisional and burn wounds [29,30]. TIMP2 has been shown to both impair [31] and accelerate cell migration in vitro [32]. In the in vitro scabs, basal *TIMP1* expression was stronger than *TIMP2* expression. Furthermore, the expression of both *TIMP1* and *TIMP2* increased with cultivation time. In contrast to the expression of *MMP9*, alterations in TGF-β signaling had no significant effect on the expression of *TIMP1* and *TIMP2*. Considering their interaction within the system, the strongest proteolytic activity was expected in the TGF-β1 and the Alk5i group, represented by the highest *MMP9*:*TIMP1* ratios. This could explain the observation that in vitro scabs stimulated with TGF-β1 remained large in size over the 14 days of culture while their stability seemed weakened. However, to verify this observation, a quantification of the scabs’ stiffness would be required. The missing resolution of the in vitro scabs stimulated with TGF-β is in line with the observation that prolonged expression of *TGF-β1* by keratinocytes and immune cells results in a chronification of the wound [33]. These results may be different when incorporating fibroblasts instead of HaCaT cells within the scab model, as excessive activation of TGF-β in wound fibroblasts is associated with keloid formation [20].

## 5. Conclusions

In summary, the here developed in vitro scab model can be cultured and analyzed for at least 2 weeks, which includes the first phases of normal wound healing. The release and expression of TGF-β and associated target genes in the in vitro scabs meet the expectations from normal wound healing. By modifying TGF-β signaling, characteristic changes that can be linked to disturbed wound healing have been observed. Thus, this model can be used to systematically investigate the mechanisms of early wound healing in vitro.

## Figures and Tables

**Table 1:** Specificities of primers used for the analysis of gene expression with F: Forward and R: Reverse primers.

Primer	Forward Sequence	Reverse Sequence	cDNA	T_A_	Cycles	Size
*18s*	GGACAGGATTGACAGATTGAT	AGTCTCGTTCGTTATCGGAAT	20 ng	56 °C	25	111 bp
*RPL13a*	AAGTACCAGGCAGTGACAG	CCTGTTTCCGTAGCCTCATG	20 ng	56 °C	30	100 bp
*TGF-β1*	TCCGGACCAGCCCTCGGGAG	CGGTCGCGGGTGCTGTTGTA	20 ng	58 °C	35	680 bp
*TGF-β2*	GCAGGTATTGATGGCACCTC	AGGCAGCAATTATCCTGCAC	20 ng	58 °C	35	206 bp
*TGF-β3*	CAGCTGCCTTGCCACCCCTC	TGCAGCCTTCCTCCCTCTCCC	20 ng	58 °C	40	601 bp
*CTGF*	CCAATGACAACGCCTCCTG	TGGTGCAGCCAGAAAGCTC	40 ng	62 °C	35	159 bp
*FN1*	CCCCATTCCAGGACACTTCTG	GCCCACGGTAACAACCTCTT	20 ng	60 °C	35	203 bp
*COL1A1*	CAGCCGCTTCACCTACAGC	TTTTGTATTCAATCACTGTCTTGCC	40 ng	60 °C	35	83 bp
*MMP1*	CCCAGCGACTCTAGAAACACA	TCTTGGCAAATCTGGCGTGT	40 ng	60 °C	35	322 bp
*MMP2*	AACATACAAAGGGATTGCCAGGA	TTCAGCACAAACAGGTTGCAG	40 ng	60 °C	35	117 bp
*MMP9*	TCTATGGTCCTCGCCCTGAA	CATCGTCCACCGGACTCAAA	40 ng	64 °C	35	219 bp
*MMP13*	CCTGCTGGCTCATGCTTTTC	AGACCTAAGGAGTGGCCGAA	40 ng	64 °C	40	141 bp
*TIMP1*	AGTTTTGTGGCTCCCTGGAA	AAGCCCTTTTCAGAGCCTTG	40 ng	60 °C	35	179 bp
*TIMP2*	ATGCAGATGTAGTGATCAGGGC	GTGATGTGCATCTTGCCGTC	40 ng	60 °C	35	256 bp
*Smad7*	TTCGGACAACAAGAGTCAGC	AAGCCTTGATGGAGAAACC	20 ng	60 °C	40	200 bp

## Data Availability

The datasets generated during and/or analyzed during the current study are available from the corresponding author on reasonable request.
